# An integrated -omics analysis of the epigenetic landscape of gene expression in human blood cells

**DOI:** 10.1186/s12864-018-4842-3

**Published:** 2018-06-19

**Authors:** Elizabeth M. Kennedy, George N. Goehring, Michael H. Nichols, Chloe Robins, Divya Mehta, Torsten Klengel, Eleazar Eskin, Alicia K. Smith, Karen N. Conneely

**Affiliations:** 1 Genetics and Molecular Biology Program, Emory University, Atlanta, GA USA; 2 Department of Human Genetics, Emory University School of Medicine, Atlanta, GA USA; 3 Department of Biology, Emory University, Atlanta, GA USA; 4 Population Biology, Ecology and Evolution Program, Emory University, Atlanta, GA USA; 5 School of Psychology and Counseling, Faculty of Health, Institute of Health and Biomedical Innovation, Queensland University of Technology, Kelvin Grove, Australia; 6 Department of Psychiatry, McLean Hospital, Harvard Medical School, Belmont, MA USA; 7 Department of Computer Science, University of California, Los Angeles, CA USA; 8 Department of Gynecology and Obstetrics, Emory University School of Medicine, Atlanta, GA USA; 9 Department of Psychiatry and Behavioral Sciences, Emory University School of Medicine, Atlanta, GA USA

**Keywords:** DNA methylation, Gene expression, Transcriptional regulation, Blood cells

## Abstract

**Background:**

Gene expression can be influenced by DNA methylation 1) distally, at regulatory elements such as enhancers, as well as 2) proximally, at promoters. Our current understanding of the influence of distal DNA methylation changes on gene expression patterns is incomplete. Here, we characterize genome-wide methylation and expression patterns for ~ 13 k genes to explore how DNA methylation interacts with gene expression, throughout the genome.

**Results:**

We used a linear mixed model framework to assess the correlation of DNA methylation at ~ 400 k CpGs with gene expression changes at ~ 13 k transcripts in two independent datasets from human blood cells. Among CpGs at which methylation significantly associates with transcription (eCpGs), > 50% are distal (> 50 kb) or trans (different chromosome) to the correlated gene. Many eCpG-transcript pairs are consistent between studies and ~ 90% of neighboring eCpGs associate with the same gene, within studies. We find that enhancers (*P* < 5e-18) and microRNA genes (*P* = 9e-3) are overrepresented among trans eCpGs, and insulators and long intergenic non-coding RNAs are enriched among cis and distal eCpGs. Intragenic-eCpG-transcript correlations are negative in 60–70% of occurrences and are enriched for annotated gene promoters and enhancers (*P* < 0.002), highlighting the importance of intragenic regulation. Gene Ontology analysis indicates that trans eCpGs are enriched for transcription factor genes and chromatin modifiers, suggesting that some trans eCpGs represent the influence of gene networks and higher-order transcriptional control.

**Conclusions:**

This work sheds new light on the interplay between epigenetic changes and gene expression, and provides useful data for mining biologically-relevant results from epigenome-wide association studies.

**Electronic supplementary material:**

The online version of this article (10.1186/s12864-018-4842-3) contains supplementary material, which is available to authorized users.

## Background

DNA methylation at CG dinucleotides (CpGs) is an essential epigenetic mechanism for many organisms. Regions of CpG-rich sequences, termed CpG islands, are found throughout the human genome. These CpG islands overlap with promoter regions or transcription factor binding sites for approximately half of mammalian genes, including nearly all housekeeping genes [1]. Canonically, methylation in promoter CpG islands inhibits the initiation of gene transcription [2]. Through modulation of gene transcription and expression, epigenetic modifications allow for morphologically distinct cell types to form from a single genome [3, 4]. Epigenome-wide association studies (EWAS) have also linked certain DNA methylation patterns to environmental factors, aging, and disease [5–14].

Unfortunately, despite a growing number of EWAS, we are still far from understanding how epigenetic changes contribute to the onset of complex diseases [2, 15]. EWAS often return large sets of marginally significant or near-significant results, many of which lie outside of defined genomic regions (i.e. genes) [16, 17]. Inferring a functional consequence of such results is difficult because our understanding of the role of methylation in gene expression is incomplete. This is especially true for EWAS hits outside promoters, as the role of DNA methylation in these regions is not fully defined [2].

Recent studies have set out to clarify the role of DNA methylation in gene expression by investigating associations between gene expression and the methylation of nearby CpGs. CpGs with methylation changes that associate with expression changes are called expression-associated CpGs, or eCpGs. The results of these studies suggest that gene transcription can be influenced by DNA methylation at CpGs that are far (> 50 kb or on a different chromosome) from the gene promoter [18–22]. Additionally, many of these studies report that changes to CpG methylation in enhancers may be central to epigenetic gene regulation. However, most of these studies tested only for eCpGs within a limited distance from each gene [18, 21–23], with few seeking to identify genome-wide eCpGs for each gene [19, 20]. In this study, we define genome-wide epigenetic signatures for more than 13 k transcripts, based on methylation at over 420 k individual CpGs in two human studies. We find evidence that CpG methylation changes associate with gene expression at great distances throughout the genome. Our results broaden the understanding of epigenetics and gene regulation and have the potential to provide critical biological insight for new and existing EWAS.

## Results

### Summary of cohorts and data

We analyzed genome-wide DNA methylomic and transcriptomic data from two cohorts. In the Grady Trauma Project (GTP), whole blood samples were collected from 333 participants (76% female) aged 18–78 years (GEO accession numbers GSE72680, GSE58137). In the Multi-Ethnic Study of Atherosclerosis (MESA), relevant data were available for purified monocytes from 1202 participants (51% female) aged 55–94 years (GEO accession number GSE56047, Table 1).

**Table 1:** Cohorts and Data for GTP and MESA

	GTP	MESA
Participants	333	1202
Tissue	Whole blood	Monocytes
Original study phenotype	Post traumatic stress disorder	Atherosclerosis
Methylation technology	Infinium HumanMethylation450 BeadChip	
Expression technology	Illumina HumanHT-12 Expression BeadChip	
Methylation probes included	472,199	422,016
Expression probes included	13,933	19,445

For both GTP and MESA, methylation data for > 480 k individual CpGs were generated from the Infinium HumanMethylation450 BeadChip (Illumina, San Diego, CA), and RNA transcript levels for > 25,000 annotated genes were quantified via Illumina HumanHT-12 v3.0 and v4.0 Expression BeadChip (see “Methods” for details).

Although both studies derive data from blood cells, GTP derives data from whole blood samples, while MESA derives data from purified monocytes (a small component of whole blood cells; see Materials and Methods). As such, we analyze both studies in parallel and make comparisons between the two, but they are not meant to be biological replicates.

### General landscape of DNA methylomic profile

We identified 1687 and 16,327 eCpGs in GTP and MESA respectively (GTP: -53 < *T* < 70, 9.7e-197 < *p* < 1e-11; MESA: -70 < *T* < 54, 1e-321 < *p* < 1e-11). These eCpGs associate with 533 and 3269 transcripts, making a total of 2466 and 34,518 unique eCpG-transcript pairs for GTP and MESA, respectively (Table 2). The discrepancy in the number of findings between GTP and MESA is likely due to power differences; with *n* = 333 for GTP and *n* = 1202 for MESA and an *α-*level of 1e-11, the studies have 80% power to detect associations where the eCpG explains as little as 16% (GTP) or 4.7% (MESA) of variation in expression. Another factor that may contribute to the discrepancy is that monocytes have a slightly larger dynamic methylation range than the predominant cell type in whole blood [24]. The average number of eCpGs per transcript was 4.6 and 11 for GTP and MESA, respectively. The median number of eCpGs per transcript was two for both GTP and MESA. eCpG-transcript pairs with associated statistics and UCSC Genome Browser tracks are provided for both GTP and MESA (see Additional files 1, 2, 3 and 4).

**Table 2:** Significant* eCpG-transcript associations for GTP and MESA

Study		GTP			MESA	
Number of eCpGs		1692			16,356	
Number of transcripts		537			3277	
eCpG-transcript pairs		2466			34,518	
Transcript pair status	Cis	Distal	Trans	Cis	Distal	Trans
Total pairs	1167	341	958	7246	3460	23,812
Positively correlated	389	114	228	2560	1578	11,985
Negatively correlated	778	227	730	4686	1882	11,827

*P ≤10^-11^

**Fig. 1:**
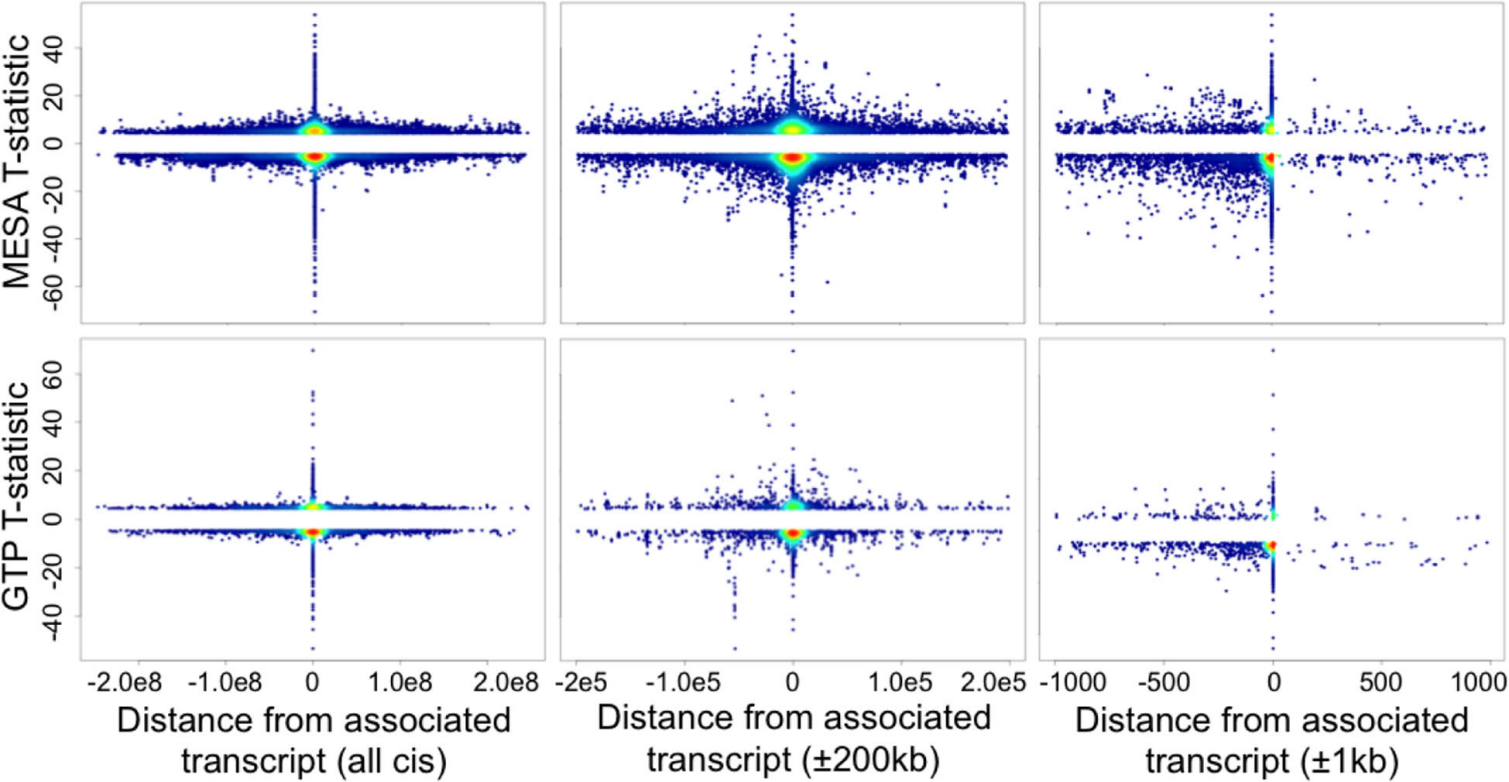
Scatter plot of T-statistic vs. distance from associated transcript, among suggestively significant eCpGs (*P* < 1e-5). The top row is from MESA and the bottom from GTP. The leftmost column is for all cis and distal eCpGs. The middle and right columns contain only eCpGs within 200 kb and 1 kb from their cognate transcript, respectively

**Fig. 2:**
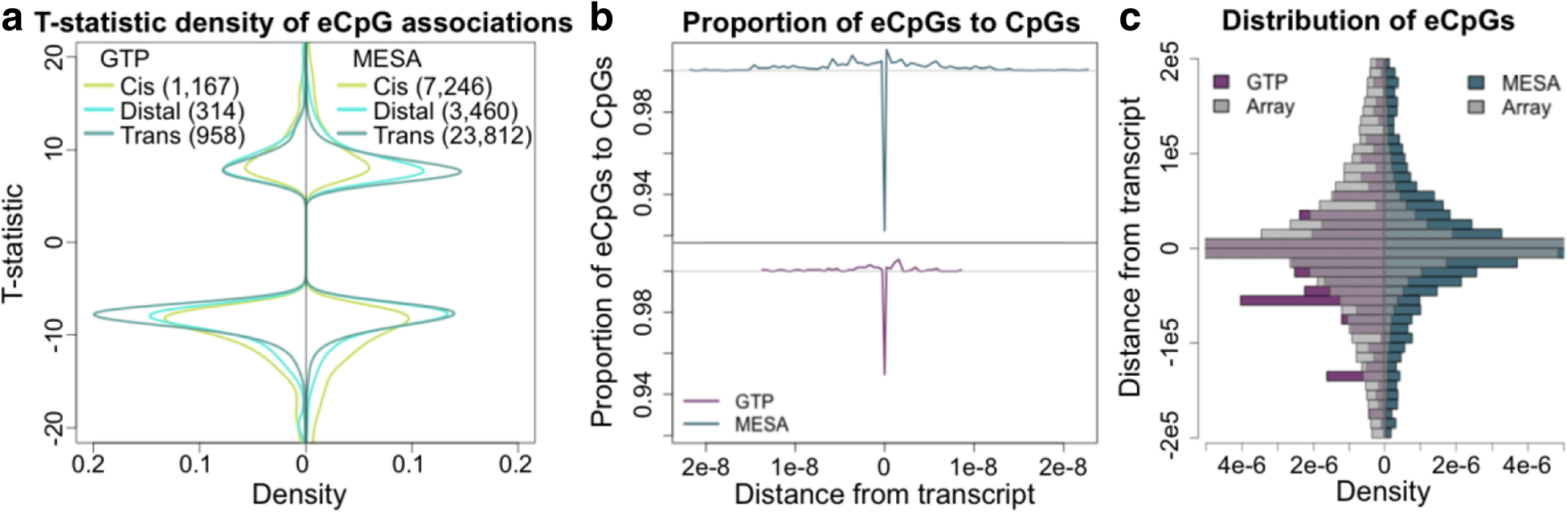
Distribution of genome-wide significant eCpGs (*P* < 1e-11). More negative than positive associations are seen in both studies (**a**). The proportion of CpGs that are eCpGs rises near genes, but drops very near and in their associated genes (**b**). eCpGs are found distal to their associated genes (**c**)

Correlations between methylation and expression were often negative in both GTP (70%, *n* = 2466) and MESA (53%, *n* = 34,518; Figs. 1, 2a; Table 2). For both GTP and MESA, there are more negatively than positively correlated eCpGs among both cis and distal eCpG-transcript pairs. However, while GTP trans eCpGs are enriched for negative correlations (OR = 1.6, *P* = 3.9e-7), MESA trans eCpGs are enriched for positive eCpG-transcript pair correlations (OR = 1.6, *P* < 2.2e-16; Table 2).

The CpGs assayed within GTP and MESA displayed the expected bimodal distribution of average methylation values, indicating that most CpGs were either fully methylated or unmethylated. In contrast, eCpGs were more likely to be intermediately methylated, with average β-values between 0.2 and 0.8 (OR = 3.6 (MESA), 3.04 (GTP), Fisher’s exact *P* < 2.2e-16 for both MESA and GTP). However, this relationship likely reflects increased power due to increased variability among intermediately methylated CpGs (Additional file 5: Figure S1).

#### Distribution of eCpGs relative to the 450 K array

When on the same chromosome (cis and distal), eCpGs were located in the associated gene or within 2500 bp of its TSS 49% (*n* = 1508) and 41% (*n* = 10,706) of the time, for GTP and MESA, respectively. However, we find that the relative proportion of eCpGs ([number eCpGs per bin/total number eCpGs] / [number CpGs per bin/total number CpGs]) increases with proximity to the associated transcript, but drops very near and in the transcript (Fig. 2b). Accordingly, the proportion of eCpGs distal to their associated (or cognate) gene exceeds the proportion of CpGs on the array that are distal to the closest transcript (for CpGs and transcripts passing QC in each study; Fig. 2c). There also appears to be a predominance of eCpGs located upstream of their associated gene (Fig. 1, third column); however this imbalance reflects the composition of the Human-Methylation450 array (Additional file 5: Figure S2).

#### Distribution of eCpGs relative to associated genes

**Fig. 3:**
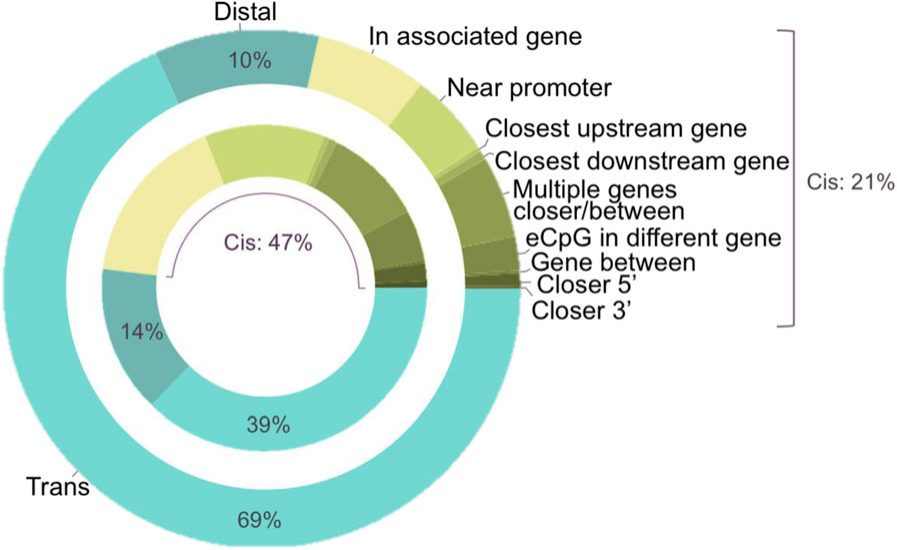
Genome-wide significant (*P* < 1e-11) eCpG-transcript relationship proportions in GTP (inner; *n* = 2466) and MESA (outer; *n* = 34,518). The green sections represent eCpGs that are < 50 kb from their associated transcript (cis); yellow represents eCpGs that fall within the gene body of their associated transcript; dark blue represents eCpGs that were < 50 kb, but on the same chromosome (distal) as the associated transcript; and light blue represents eCpGs that were on a different chromosome from the associated transcript (trans). Definitions of each category are given in Fig. 4 and Materials and Methods section

**Fig. 4:**
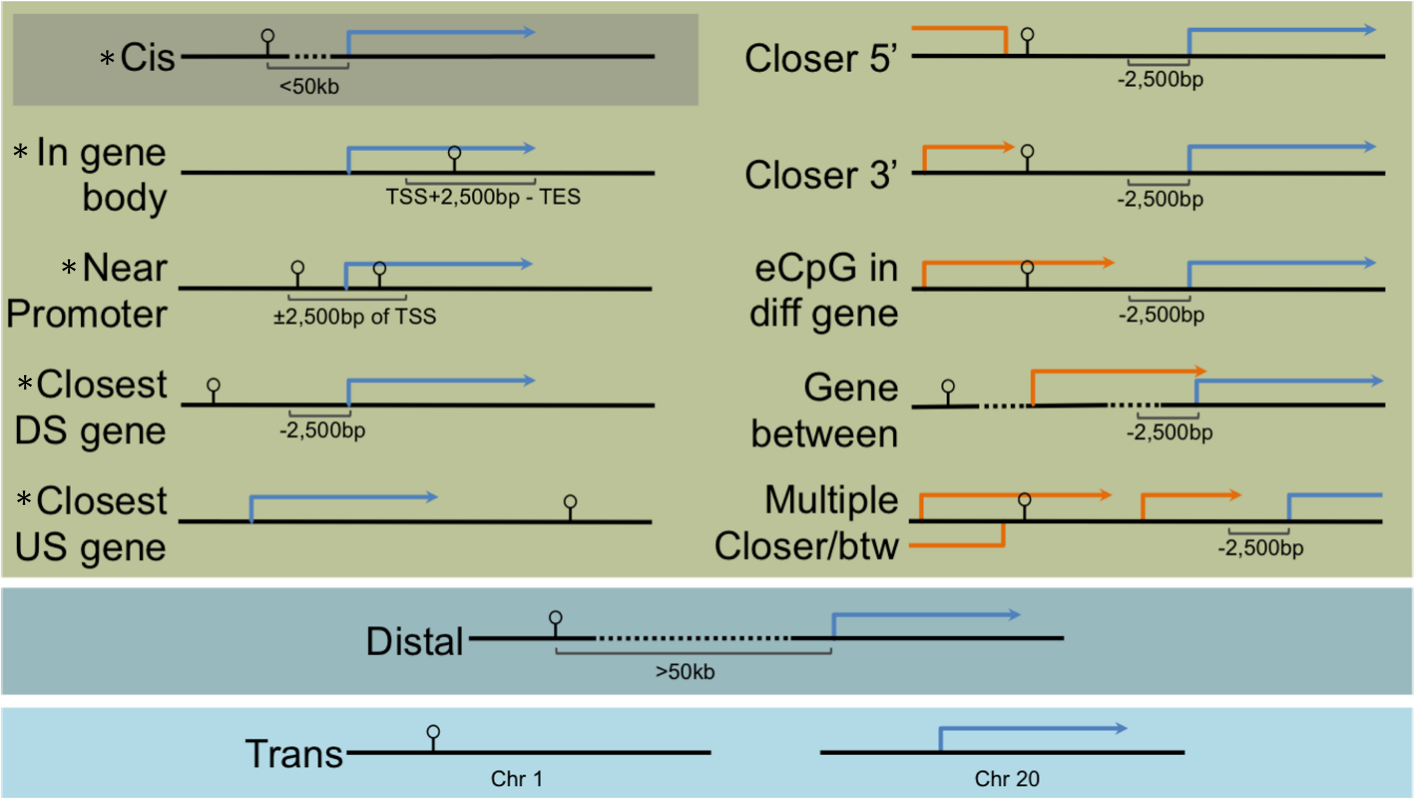
Graphical examples of each functional category are shown for positive strand eCpG (stick with open circle) and transcript (blue arrow) pair associations. Blue arrows represent the gene transcription area (TSS-TES) that was annotated to the expression probe in the eCpG-transcript pair by overlap with a refseq or ensemble exon. Orange arrows represent examples of other annotated genes that are near the eCpG-transcript pair. DS is downstream, US is upstream, TSS is transcription start site, and TES is transcription end site. * indicates canonical methylation-expression roles.

In GTP and MESA, distal and trans eCpGs constitute 53% (*n* = 2466) and 79% (*n* = 34,518) of eCpGs, respectively (Fig. 3, Table 2, Additional file 5: Table S1), indicating that eCpGs are not primarily near associated genes. Figure 4 defines the possible eCpG-transcript pair scenarios, relative to the gene annotated to the transcript and other nearby genes, described further in Materials and Methods. In short, we consider canonical eCpG-transcript pairs to be those in which the eCpG is within the gene or within 2500 bp of the gene’s TSS, or the associated gene is the closest gene to the eCpG. Among cis eCpGs, nearly 35% do not conform to a canonical methylation-expression role where the eCpG associates with the nearest gene (GTP *n* = 1167; MESA *n* = 7246; Fig. 3). Canonical eCpG-transcript pairs are captured in the remaining 65% of cis eCpGs (GTP *n* = 1167; MESA *n* = 7246; 21 to 48% of all eCpGs; GTP *n* = 2466; MESA *n* = 34,518; see Fig. 3).

### Corroboration of eCpG results

#### Between study comparison

To corroborate the eCpGs identified here, we compared eCpG-transcript pairs across studies. Among eCpG-transcript pairs significant in GTP, 44% (*n* = 1260) of cis pairs (53% of promoter eCpG-transcript pairs, *n* = 383), 30% of distal pairs (*n* = 341) and 27% trans (*n* = 958) pairs are significant in MESA. Randomly permuting the transcript IDs among the significant eCpG-transcript pairs from both studies and repeating the calculation 10,000 times yielded no higher than 3% of GTP distal- and trans- pairs occurring in MESA distal- and trans- pairs.

#### Within study comparison

**Fig. 5:**
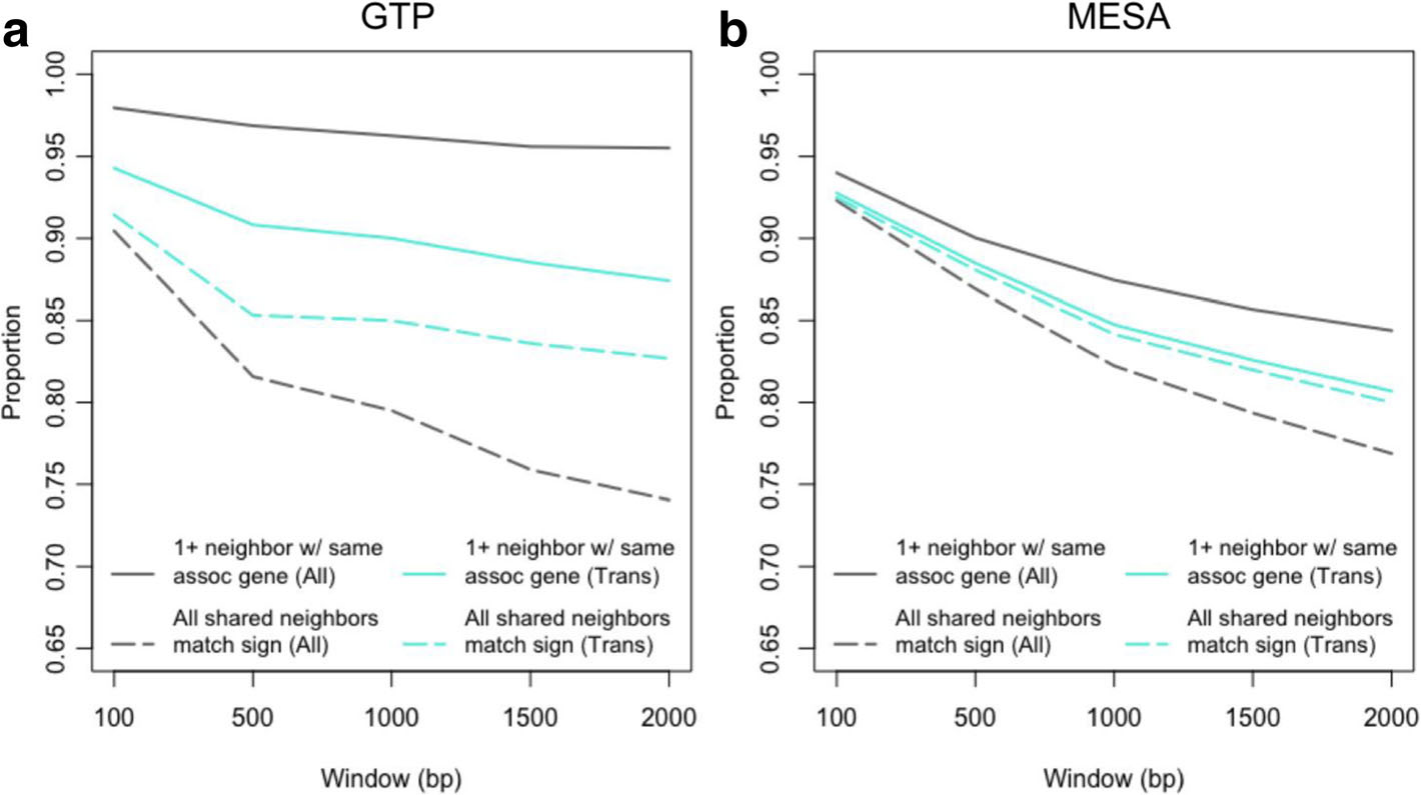
Shared gene associations among neighboring eCpGs. Proportion of proximal eCpGs (neighbors) in GTP and MESA with the same associated gene or same associated gene and direction of association as the query CpG. Neighbors were located within the specified window size on either side of the query CpG. Associated gene overlap among proximal eCpGs appears to be a function of distance. The majority of neighboring eCpGs sharing an associated gene, associate with the gene in the same direction

To corroborate our eCpGs within each study, we examined associated gene congruence among neighboring eCpGs, under the assumption that neighboring eCpGs should associate with expression of the same transcript. This assumption is supported by research suggesting that methylation patterns between neighboring sites are correlated [20, 25], and that groups of CpGs, as opposed to individual CpGs, may be important in gene regulation [26, 27]. Among eCpGs having a neighbor within 500 bp, 97% (GTP) and 90% (MESA) have a neighbor significantly associated with at least one of the same cognate genes, 86% (GTP) and 89% (MESA) have at least one neighbor that is consistent with regard to direction of correlation and 82% (GTP) and 87% (MESA) have completely congruent neighbors (GTP *n* = 738, MESA *n* = 6290; Fig. 5). Trans eCpGs have a slightly lower proportion of neighbors significantly associated with the same cognate gene (GTP = 91%, MESA = 88%), but 85% of GTP and 88% of MESA eCpGs have all neighbors of congruent direction (GTP *n* = 109, MESA *n* = 3074). The proportion of proximal CpG neighbors with matching associated gene and sign predictably declines with increasing window size (Fig. 5).

### Functional analysis of eCpGs

#### Functional trends among all eCpGs

**Fig. 6:**
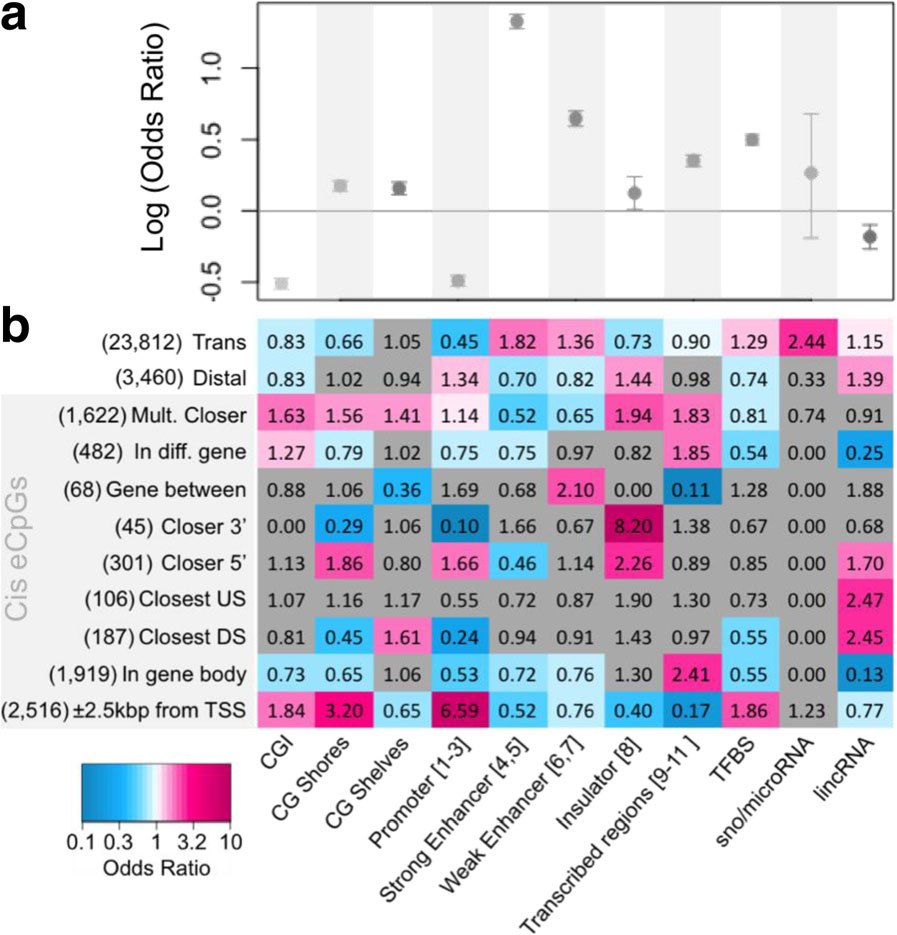
Enrichment of chromatin features among eCpGs. **a**) Enrichment of eCpGs (odds ratios and 95% confidence intervals) for the listed chromatin features, among all CpGs tested in MESA (*N* = 422,016). **b**) Enrichment of eCpGs for the listed chromatin features, among genome-wide significant eCpGs in MESA (*N* = 34,518). Shaded categories are cis. Blue indicates significant depletion and red, significant enrichment (*P* < 0.05). Bracketed numbers in the chromatin features indicate the ChromHMM state. Numbers in parentheses indicate the number of eCpGs in the category. Definitions: Left: Given in Fig. 4 and Materials and Methods Bottom: “CGI” are CpG islands. “TFBS” is transcription factor binding site

Next we used publicly available data to assess functional trends among eCpGs [28–30]. As part of the ENCODE project [30] Ernst, et al. (2011) used a hidden Markov model to partition the genome into functional domains based on ChIP-seq data for histone modifications, RNA polymerase occupancy, and other chromatin features. We used the resulting data set, called ChromHMM, along with CpG island, long intergenic non-coding RNA (lincRNA), transcription factor binding site (TFBS) and small nucleolar and microRNA (sno/microRNA) genomic intervals to evaluate the chromatin structure surrounding eCpGs [28–30]. When considering all CpGs tested in MESA, genome-wide significant eCpGs are depleted among CpG islands (CGI; OR = 0.60, *P* = 7.5e-170) and promoters (ChromHMM states 1–3; OR = 0.55, *P* = 1.4e-168), but enriched among the more variable CpG shore (1500 bp out from CGI; OR = 1.2, *P* = 2.1e-22) and shelf (1500 bp out from CG shores; OR = 1.2, *P* = 2.6e-12) regions (Fig. 6a). We also find that eCpGs are enriched among transcription factor binding sites (TFBS) and highly enriched among annotated enhancer regions (ChromHMM states 4–7; Fig. 6a; OR > 1.9 and *P* < 2.2e-16). GTP shows a similar enrichment for enhancer regions (top row in Additional file 5: Figure S3). This result is consistent with other studies that have found a significant enrichment of eCpGs among enhancers [18, 23].

#### Functional trends among trans eCpGs

When assessing the enrichment of chromatin states among the various categories of significant eCpGs, we find that trans eCpGs are enriched among both strong (ChromHMM states 4 and 5; OR = 1.8, *P* = 2.2e-72) and weak (ChromHMM states 6 and 7; OR = 1.4, *P* = 5.4e-18) enhancer annotations (Fig. 6b). Like the overall pattern, we see that trans eCpGs are depleted among CGI (OR = 0.83, *P* = 3.6e-10) and promoters (OR = 0.45, *P* = 5.5e-168), and enriched among TFBS (OR = 1.3, *P* = 2.8e-21). Interestingly, we also observe that trans eCpGs are enriched among regions of the genome annotated as sno and microRNAs (OR = 2.4, *P* = 9.8e-3; Fig. 6b).

#### Functional trends among cis and distal eCpGs

Unlike trans eCpGs, we see that enhancers are primarily depleted among the various cis and distal eCpG categories described in Materials and Methods and Fig. 4. Additionally, insulators (ChromHMM state 8) are enriched among cis and distal eCpGs (1.4 < OR < 8.2, *P* < 0.03). Promoter (1.1 < OR < 6.6, *P* < 0.04) and CpG islands (1.3 < OR < 1.8, *P* < 0.03), shores (1.6 < OR < 3.2, *P* < 4.8e-07) and shelves (1.4 < OR < 1.6, *P* < 0.01) are more often enriched among cis eCpG categories. We also see a strong enrichment of cis (1.7 < OR < 2.5, *P* < 0.05) and distal (OR = 1.4, *P* = 5e-4) eCpGs among regions of the genome annotated as lincRNAs. We note a depletion of enhancers in the cis categories in which lincRNA eCpGs are enriched (OR = 0.5, *P* = 5.9e-05; Fig. 6b).

#### Gene ontology analysis

We used GO to assess molecular function terms among all eCpGs, cis and distal eCpGs and trans eCpGs, as well as among transcripts associated with trans eCpG methylation. We found that eCpGs are enriched for nucleotide binding molecular functions, like sequence specific DNA binding (OR = 2.6, *P* = 1.2e-04) and transcription factor binding (OR = 4.3, *P* = 2.6e-04). DNA-binding and transcription factor molecular functions are also enriched in cis/distal and trans eCpGs (1.5 < OR < 4.2, *P* < 1.8e-04). Finally, transcripts that associate with trans eCpG methylation were enriched for chromatin readers, writers (1.7 < OR < 3.8, *P* < 6.3e-04) and transcription co-activator genes (Ligand-dependent nuclear receptor transcription coactivator activity OR = 2.9, *P* = 1.2e-03; Additional file 5: Tables S2-S5). All *p*-values listed above correspond with a false discovery rate (FDR) < 0.05.

### Analysis of gene body eCpGs

**Fig. 7:**
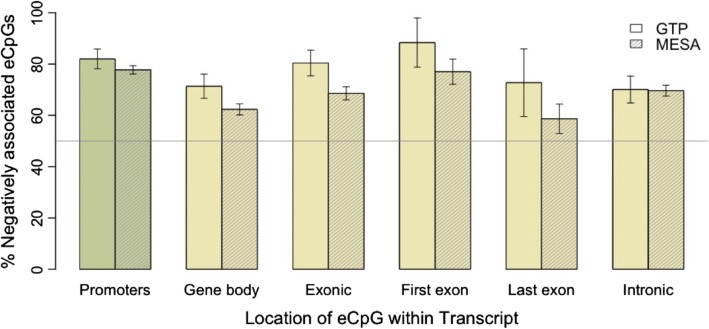
Negative eCpG-transcript correlations in GTP and MESA. The fraction of negative eCpG-transcript associations is greater than 50% in promoters and gene bodies. More negative associations are found in the first exon than the last

It has been frequently reported that DNA methylation is negatively correlated with gene expression in promoters, but positively correlated with gene expression within gene bodies [2, 18, 21]. Here, we observe that DNA methylation is negatively correlated with transcript expression the majority of the time, in any location (Fig. 7). Among significant eCpGs in MESA, negative correlations are enriched among gene body eCpGs (OR = 1.5, *P* = 2.6e-16). Among significant eCpG-transcript associations where the CpG was located within the gene body of its associated transcript, 1) the correlation was negative 71% (*n* = 356) and 62% (*n* = 1919) of the time, for GTP and MESA, respectively, 2) the direction of correlation was consistent across multiple eCpGs within a single transcript 85% (*n* = 87; GTP) and 72% (*n* = 601; MESA) of the time, and 3) among transcripts with consistent associations across multiple eCpGs, the correlations were negative 81% (*n* = 74; GTP) and 76% (*n* = 434; MESA) of the time. Among CpGs within the first and last exon of their associated transcript, we note that although still primarily negative, fewer eCpGs are negatively correlated with transcript expression in the last exon (59% in MESA, 73% in GTP), in comparison to the first exon (77% in MESA, 87% in GTP) (Fig. 7).

#### Functional trends among gene body eCpGs that negatively correlate with expression

One hypothesis that attempts to account for an excess of negative correlations among gene body eCpGs posits that these eCpGs are found in intragenic regulatory elements like promoters and enhancers located within the gene they control [31, 32]. We observe a slight enrichment of annotated promoters among negatively correlated gene body eCpGs (OR = 1.8, *P* = 0.002). An even stronger enrichment of negatively correlated gene body eCpGs among annotated enhancer regions (OR = 2.2, *P* < 2.2e-16) suggests that transcriptional regulators within gene bodies may be important to gene regulation.

## Discussion

EWAS often identify CpGs that lie outside of defined genomic regions like promoters, which are typically considered the canonical target for epigenetic gene repression [2, 16, 17]. Inferring a functional consequence for these CpGs is difficult because our understanding of the role of methylation in regulation of gene expression and disease is incomplete. We find that the majority of eCpGs do not conform to canonical methylation-expression roles. Our results highlight a shortcoming of current CpG functional annotation, as these non-canonical methylation-expression relationships would be incorrectly assigned to the nearest gene in EWAS interpretation.

We find that many eCpG-transcript pairs are consistent between studies and that neighboring eCpGs within studies tend to correlate with the same gene. Although it is encouraging to find matching pairs between studies, it is unsurprising that there is not complete overlap given differences in both power and cell type and ethnic background across studies. GTP is a relatively small study, whose data were derived from whole blood in an African American cohort. MESA, a much larger study from a cohort of mixed ethnicity, derived data from monocytes, which only account for a small proportion of whole blood cells, on average. As such, MESA and GTP are not intended to be replicates but a comparison across whole blood and monocytes. In a study of cis CpG-transcript associations, Liu et al. (2013) found that few observed expression-associated methylation sites were specific to any ethnic category, so it is unlikely that differences between eCpGs found in GTP and MESA are driven by ethnic composition. Our results suggest that our eCpGs represent robust associations that are consistent between neighboring CpGs and across datasets.

Among transcripts passing QC (GTP: 13,933, MESA: 19,445), only 3.8% of GTP transcripts and 17% of MESA transcripts significantly correlated with CpG methylation. Because the two studies are powered to detect associations explaining > 16% or > 4.7% of variance in expression, respectively, eCpG-transcript associations with subtler correlations would not have been detected. It is possible that in many cases either the transcripts or the CpGs passing QC were not variable enough in the tissues studied to detect associations, or that some of the genes are not epigenetically regulated in blood. This hypothesis is supported by the observation that in MESA, which is powered to detect subtler associations than GTP, the average variance in methylation β-values for identified eCpGs was lower (3.6e-03) than for eCpGs identified in GTP (6.4e-03), while variation in non-eCpGs was similar across both datasets (Additional file 5: Figure S1). Finally, the variance in some genes could be due to factors other than CpG methylation, for instance, regulation by other genes or higher-level chromatin mark (i.e. histone modifications).

Our enrichment and gene ontology results make the case for a complex network of epigenetic control. In addition to the more canonical promoter eCpGs that associate with proximal gene expression, we also see that eCpGs associate with gene expression distally, through enhancers, insulators and long intergenic non-coding RNAs (lincRNAs). Importantly, we find that enhancer elements, micro and small nucleolar RNAs are prominent among eCpGs that correlate with the expression of genes on different chromosomes (trans). The GO analysis suggests that for each gene, we have likely constructed a regulatory profile that encompasses the indirect, trans effects (which could include regulatory networks) as well as direct, cis effects (including cis and distal DNA methylation). Because we find many eCpGs, genome-wide, that associate with transcription factor genes and chromatin modifiers, our results may include scenarios in which gene expression influences DNA methylation patterns, as well as vice-versa [33]. Although these findings represent associations and do not provide information on causality, they could prove useful in annotating EWAS results for CpGs with potential roles in regulatory networks.

Overall, our results indicate that CpG methylation interacts with gene expression primarily through enhancer CpGs, rather than promoter CpGs. Enhancers, as distal regulatory elements, are methylation sensitive transcription factor binding sites that promote tissue-specific gene expression [2, 3]. Other studies have also noted an enrichment of enhancer regions among eCpGs [18, 23]. One proposed model of gene regulation suggests that promoter methylation is relatively static, having either a restrictive (hypermethylated) state, or permissive (hypomethylated) state at which dynamic enhancer methylation modulates gene expression levels [18]. In this scenario, promoter eCpGs are far less likely than enhancer eCpGs to be identified due to their low variability [23]. Our results support the important role of enhancer CpG methylation in epigenetic gene regulation, but expand on this model to suggest that enhancer methylation can correlate with gene expression changes on other chromosomes.

We also find that insulator eCpG methylation plays a prominent role in cis and distal gene expression. Insulators are thought to promote gene expression by bringing enhancers and promoters into close proximity through the binding of the CCCTC-binding factor (CTCF), which can dimerize to form stable chromatin loops [34, 35]. The binding affinity of CTCF to insulator sequences is influenced by DNA methylation [36]. Here we see that insulators are enriched among cis and distal eCpGs. Currently, the resolution of HiC, a method to detect chromatin loops, does not allow us to confidently discern the significance of eCpG-transcript interactions, compared to CpGs of similar location and functional annotation. However, in a comparison of distributions between eCpG-transcript distances and HiC DNA looping interaction distances, we found that eCpG-transcript frequencies decrease as a function of distance (Fig. S4, green), at a similar rate to the DNA looping frequencies seen in HiC data (Additional file 5: Figure S4, blue, and supplemental methods in Additional file 6) [35]. Overall our results support the role of insulators in regulation of gene expression, potentially through the formation of functional DNA loops involving enhancer and insulator elements.

MicroRNAs regulate more than 50% of mRNAs [37] and are in turn regulated by DNA methylation [38, 39]. We see a strong enrichment of trans eCpGs among micro/snoRNAs, so it is intriguing to speculate that trans eCpG-transcript associations are due, at least in part, to post-transcriptional regulation by microRNAs. We also see that cis and distal eCpGs are enriched among lincRNAs. Evidence suggests that lincRNAs play an important role in gene expression, particularly as eRNAs (enhancer RNAs), which are RNAs transcribed from enhancer sequences and may act as scaffolding for DNA looping or co-activator recruitment to a gene promoter [34]. Interestingly, the enhancers that give rise to eRNAs are distinct from enhancers that act as transcription factor binding sequences [40]. In our results, we also see a depletion of enhancers in the cis categories in which lincRNA eCpGs are enriched. From our results, we propose that DNA methylation may be a key player in cis, distal and trans transcriptional control through the action of non-coding RNAs.

Our study finds that most eCpG-transcript correlations are negative, even among gene bodies. Our findings are in line with other studies that report the predominance of negative correlations [19–23]. The primary difference between studies that find mostly negative methylation-expression correlations and those that find negative correlations in promoters and positive correlations in gene bodies is study design. Most studies finding positive gene body correlations were considering the correlation of expression and methylation across all genes in a single genome [18, 41, 42]. In contrast, the majority of studies finding negative correlations in gene bodies were considering correlation of expression and methylation across individuals, separately for each CpG [21, 23]. A within-genome comparison observing that more highly expressed genes tend to show hypermethylation within gene bodies is simply a comparison of different genes and does not speak to the effect of changes in DNA methylation at any particular gene. In general, studies that assess DNA methylation in gene bodies across individuals find that, most of the time, increases in DNA methylation are associated with decreases in gene expression [19–23].

We also explore the potential role of intragenic DNA methylation. We provide evidence here that negatively correlated gene-body eCpGs are often the result of intragenic regulatory elements (e.g. promoters and enhancers). An alternative hypothesis states that positive correlations between CpG methylation and gene expression are the result of overlapping genes/variants [43, 44]. We only found five instances in our data in which one eCpG was associated with an overlapping set of genes (in the promoter of one and the gene body of the other). While five examples are insufficient to draw conclusions, the majority of these CpGs correlated negatively from the promoter, and positively from the gene body, suggesting that positive gene body methylation correlations could result from the anticorrelation of the gene expression itself (Additional file 5: Table S6 and supplemental methods in Additional file 6). Neither of these hypotheses fully explain the occurrence of either positive or negative eCpG correlations within gene bodies. Rather, they suggest that there is no all-encompassing biological truth to these associations.

## Conclusions

We have characterized the genome-wide DNA methylomic profile for gene expression in human blood cells. Many of our results are reproducible between whole blood and monocytes and are spatially correlated within studies. Unlike similar studies, we found that most eCpGs were very distal and trans to their associated genes. These results highlight the shortcomings of proximity based CpG annotations, as even cis eCpG-transcript associations often do not involve the closest downstream TSS. In fact, the majority of associations were distal or trans, representing a serious gap in functional annotation for epigenome-wide association studies.

Like others, we find an overabundance of enhancer eCpGs, highlighting the importance of enhancers, possibly over promoters, in gene expression variation [18, 23]. We also note enrichments of insulators and non-coding RNAs, like microRNAs and lincRNAs among eCpGs. Our results point to DNA methylation as a possible link between gene expression and higher-order chromatin organization, as well as another layer in post-transcriptional regulation.

Like studies of similar design, we find an abundance of negative CpG-transcript associations [19–23], which conflicts with earlier reports that gene body methylation positively correlates with gene expression [18, 32, 41, 45, 46]. We find some support for the hypothesis that negatively-correlated gene-body eCpGs are in annotated promoters and enhancers [32], which suggests an important role for alternate gene-body promoters and intragenic enhancers in gene expression. However, we do not find support for the presence of negative gene-body methylation associations as a result of overlapping gene expression.

Finally, our gene ontology results, like our enrichment results, portray a complex, multi-dimensional picture of epigenetic interactions in the genome. eCpGs are enriched in molecular functions like transcription factor binding and sequence specific DNA binding. Among transcripts that associate with trans eCpG methylation, we find an enrichment of chromatin readers, writers and transcription co-activator genes.

Our findings suggest that limiting our interpretation of EWAS results to the nearest gene might be short-sighted, as DNA methylation may have many indirect effects (e.g. modulating the expression of a transcription factor) that influence gene expression or vice-versa. Overall, these results broaden our understanding of the ways that CpG methylation interacts with gene expression, genome-wide, and provide data that may be useful for mining meaningful biological insights from EWAS.

## Methods

### Data preprocessing and QC

The Grady Trauma Project (GTP) is a cross-sectional study of stress-related outcomes. Participants were recruited from the waiting rooms of Grady Memorial Hospital’s General Practice or Obstetrics and Gynecology departments in Atlanta, GA. Participants are from an inner-city population with higher than average rates of trauma exposure, but are representative of this population as they are not specifically ascertained for presence of disease or trauma. Genome-wide DNA methylation and gene expression measurements were generated for 333 human blood samples. GTP participants included in this study range between 18 and 78 years old, are 76% female and all are African-American [47].

The Multi-Ethnic Study of Atherosclerosis (MESA) is a study designed to examine cardiovascular disease. The MESA Epigenomics and Transcriptomics Study specifically investigates the association between CpG methylation and gene expression in purified human monocytes collected from the MESA population. For this study, 1202 participants were chosen randomly from samples collected between April 2010 and February 2012 from MESA field centers in Baltimore, MD; Forsyth County, NC; New York, NY; and St Paul, MN. Participants range in age from 55 to 94 years old, are 51% female, and self identified as Caucasian (47%), African American (21%), or Hispanic (32%) [23].

For both GTP and MESA, methylation data for > 480 K individual CpGs were generated from the Infinium HumanMethylation450 BeadChip (Illumina, San Diego, CA), and RNA transcript levels for > 25,000 annotated genes were quantified via Illumina HumanHT-12 v3.0 and v4.0 Expression BeadChip. We have provided a detailed description of both datasets, including sample information, data processing, QC, and normalization, in the supplemental methods (see Additional file 6). We excluded CpGs and transcripts that did not pass QC, were on the X or Y chromosomes or were poor quality. After QC, 13,933 expression probes (transcripts) and 483,399 CpG probes (CpGs) remained for GTP, and 19,445 transcripts and 422,016 CpGs remained for MESA.

### Association analysis

To model the associations between gene expression and CpG methylation at specific sites while adjusting for global expression and methylation differences between individuals, we used a linear mixed model framework developed to account for inter-individual correlation structure in expression data due to unknown confounders (inter-sample correlation emended or ICE) [48]. This method was more successful at controlling inflation than including covariates for estimated cells types via the Houseman method (Additional file 5: Figure S5) [49]. For all transcripts and CpGs in each study, we regressed log expression signals for one transcript on methylation β-values for a single CpG, while controlling for fixed effects (age and sex for GTP and age and composite race/gender/study-site for MESA) and unknown random effect covariates using ICE (eq. 1). We implemented this framework in the python program pyLMM (http://genetics.cs.ucla.edu/pylmm/) to test for association between methylation at CpG *j* and the expression level of transcript *k*, by fitting the model:

1 $$ {y}_{\mathrm{k}}={\mu}_{\mathrm{k}}+{M}_{\mathrm{j}}{a}_{\mathrm{j}\mathrm{k}}+x{\beta}_{\mathrm{j}\mathrm{k}}+{u}_{\mathrm{k}}+{\in}_{\mathrm{j}\mathrm{k}} $$

Letting *n* be the number of individuals, *y*_k_ is a vector of log expression levels at gene *k* with length *n*, *μ*_k_ is a size *n* vector denoting the mean of log expression levels over *n* individuals, *M*_j_ is a size *n* vector of methylation proportions at CpG *j*, *x* is an *n*✕2 matrix of covariates (age and sex), *u*_k_ ~ N(0, σ^2^_g_H) is a multivariate normally distributed term representing effects due to other unmeasured confounders such as cellular heterogeneity, and *ϵ*_jk_ ~N(0, σ^2^_e_I) are residual errors. I is an *n*✕*n* identity matrix and H is the *n*✕*n* intersample correlation matrix, described below.

### Intersample correlation matrix

The global intersample correlation matrix H is estimated from the expression data. Let Y be an *m*✕*n* expression matrix for *m* genes and *n* individuals. Then let Z be an *m*✕*n* matrix where each element from the *k*^th^ transcript and *l*^th^ individual *Z*_kl_ = (*y*_kl_ − *μ*_k_)/*σ*_k_; *μ*_k_ is the mean and *σ*_*k*_ is the standard deviation of log expression values of the *k*^th^ transcripts. The estimated intersample correlation matrix Ĥ, is defined as the covariance of Z, and is in eq. (1) to correct for unmeasured confounding factors.

### Analysis of results

In the association analysis, we analyzed all combinations of transcripts and CpGs, for a total of 6.6 billion comparisons for GTP and 8.2 billion comparisons for MESA. For each transcript, pyLMM generated summary statistics for the association of all CpGs. Based on these statistics, genomic inflation factors (GIF) were calculated as median (T-statistic)^2^/0.4549 for each transcript. We removed transcripts with a GIF > 2 from further analysis. We also removed CpG-transcript pairs in which the associated transcript was annotated as bad quality or as having no matching sequence in the genome [50].

A re-annotation of the Illumina HumanHT-12 v3.0 and v4.0 Expression BeadChip arrays by Barbosa-Morais and others (2010) indicates that many probes have the potential to anneal to multiple regions in the genome, by sequence homology (determined via BLAST and BLAT searches) [50]. This non-specific binding could lead to an inaccurate picture of eCpG-transcript associations, especially when the potential binding locations for an expression probe are located on multiple chromosomes. To avoid this issue, we allowed each expression probe to have multiple locations, based on the new annotation. Using the refseq and ensembl databases [51, 52], we assigned each expression probe location to a gene by overlap with an exon. We chose the location of the expression probe for each eCpG-transcript association, prioritizing expression probe locations that were closer in proximity to the eCpG, could be annotated to a gene and were listed by Barbosa-Morais as the primary > secondary > other genomic match (see supplemental methods in Additional file 6).

To establish a similar cutoff for significance across GTP and MESA, we considered CpG-transcript pairs with *p* < 10^− 5^ as suggestive and *p* < 10^− 11^ as significant. This value corresponds to Bonferroni adjustment for 5 billion independent tests, so is quite conservative given the high levels of correlation between tests. We defined CpGs that significantly associate with transcript expression as eCpGs.

We classified eCpGs, broadly, as cis (within 50 kb of associated probe), distal (greater than 50 kb from associated gene, but on the same chromosome) or trans (on a different chromosome from the associated gene). Within those broad categories, we established the following detailed classifications to describe each eCpG-transcript pair with respect to the gene the associated transcript is annotated to, as well as other nearby genes (by average refseq and ensembl gene locations (see transcript annotation in supplemental methods in Additional file 6)): trans (the eCpG was on a different chromosome than the transcript), distal (the eCpG was > 50 kb from the transcript, but on the same chromosome), in gene body (the eCpG was > 2500 bp downstream of the associated gene’s TSS and upstream of the associated gene’s TES), near promoter (the eCpG was within 2500 bp upstream or downstream of the associated gene’s TSS), closest upstream gene (the TES of the associated gene was closer to the eCpG than the next closest gene), closest downstream gene (the eCpG was not within 2500 bp of the associated gene, but the TSS of the associated gene was closer to the eCpG than the next closest gene), closer 5′ (the eCpG was farther from the associated gene’s TSS than from another gene’s TSS on the opposite side of the eCpG), closer 3′ (the eCpG was closer to the TES of another gene than the associated gene than to either the TSS or TES of the associated gene), gene between (there was another gene’s TSS between the eCpG and the associated gene’s TSS), eCpG in different gene (the eCpG was not near the promoter of the associated gene and was between the TSS and TES of another gene), multiple closer/between (the eCpG-transcript pair falls into multiple of the aforementioned cis categories; Fig. 4).

### Between study corroboration

Next we sought to find out how often GTP eCpG-transcript pairs were consistent in MESA results among the cis, distal and trans categories. To compare results between studies, we found eCpG-transcript pairs in the GTP results that were consistent in the MESA results by CpG ID, expression probe ID, expression probe location, and direction of correlation.

To compare the number of eCpG-transcript pairs found consistent across studies within the distal and trans categories to the number achieved by random chance, we re-analyzed the results 10,000 times. For each permutation, we randomly shuffled the expression probe IDs within each study and category.

### Within study corroboration

For each eCpG found in both GTP and MESA, we interrogated neighboring eCpGs within five windows extending 100, 500, 1000, 1500 and 2000 bp to each side of the query eCpG. For each window, we compared the genes associated (see transcript annotation in supplemental methods in Additional file 6) with the query eCpG to the genes associated with the neighboring eCpGs. We computed the percentage of eCpGs sharing an associated gene with a neighboring eCpG as the number of eCpGs that share at least one associated gene with at least one neighboring eCpG divided by the total number of eCpGs within the window. We then computed the percentage of eCpGs sharing at least one associated gene with the same direction of correlation with at least one neighboring eCpG. Lastly we computed the percentage of eCpGs at which all neighboring eCpGs shared both genes and direction of correlation with the query eCpG. This analysis was conducted for all eCpGs and then separately for trans eCpGs.

### Functional analysis of eCpGs

We downloaded the following datasets from the UCSC table browser for GRCh37/hg19 [30]:

1. CpG Islands
2. Broad ChromHMM for GM12878 [28]
3. Transcription factor ChIP V3 (transcription factor binding sites)

We functionally annotated eCpGs based on overlap of the CpG location with the intervals provided by UCSC for the features listed above. Additionally, CpG island shores were defined as regions extending 1.5 kb out from CpG islands and CpG island shelves were defined as regions extending 1.5 kb out from shores. Intervals for all 15 genomic states provided with the ChromHMM dataset were utilized in this annotation. We assessed these annotations, using Fisher’s exact tests, in two different ways. First we considered all CpGs tested for each study. Each CpG was only represented once (for each study) and was tested for enrichment in a functional category (e.g. CpG island, ChromHMM category) and significant eCpG status (i.e. significant vs not significant). Second, among only significant eCpG-transcript pairs, eCpG-transcript classifications (e.g. “in gene”, “closest upstream gene”; described above and in Fig. 4) were tested for enrichment among the various functional categories (e.g. CpG island, ChromHMM category). Because many CpGs associated with multiple transcripts, and vice versa, CpGs or transcripts could fall into more than one category and be present more than once in the test. However, each unique CpG-transcript pair falls into a single category and is present only once in the test.

### Gene ontology analyses

We used the R library GOstats [53] to assess enrichment of molecular function gene ontology terms among eCpGs. eCpGs that associated with a transcript with *p*-values < 10^− 5^ were included in the analysis. We applied the hypergeometric test to calculate odds ratios and p-values, and estimated the false discovery rate by the Benjamini & Hochberg method [54]. For this analysis, eCpGs that did not fall within a gene were assigned the Entrez gene ID of the gene with the closest downstream TSS. We assessed eCpGs in the following scenarios: all eCpGs, cis and distal eCpGs and trans eCpGs. Additionally, we assessed gene ontology among transcripts associated with trans eCpG methylation.

### Gene body eCpG analysis

We calculated the number of eCpGs that were negatively correlated with their cognate genes in the following categories: gene body (TSS+/− 2500 bp to TES for positive/negative strand genes), intronic, exonic, in first exon, in last exon (as determined by the average exon locations; see supplemental methods in Additional file 6).

We next address the hypothesis that negatively correlated gene body eCpGs are the result of intragenic gene regulators (e.g., promoters and enhancers). To test this hypothesis, we looked for enrichment of negatively correlated vs. positively correlated gene body eCpGs among ChromHMM annotated promoters (states 1–3) or enhancers (states 4–8).

## Additional files

Additional file 1: eCpG-transcript pairs, plus additional information for GTP. (TXT 9056 kb)

Additional file 2: eCpG-transcript pairs, plus additional information for MESA. (TXT 44022 kb)

Additional file 3: UCSC track for eCpGs from GTP. (BED 2584 kb)

Additional file 4: UCSC track for eCpGs from MESA. (BED 8181 kb)

Additional file 5: **Figure S1.** Distribution of methylation beta-value variances, plotted on a log scale. **Figure S2.** Distances between each CpG and the closest TSS for all CpG probes and expression probes included in GTP and MESA. **Figure S3.** Odds ratios for enrichment of chromatin features among CpGs. **Figure S4.** eCpG-transcript distance vs. HiC interaction frequency. **Figure S5.** Comparison of Genomic Inflation Factors obtained for GTP by the Houseman method and ICE. **Table S1.** Breakdown of eCpG-transcript status. **Table S2.** Gene Ontology enrichment among all eCpGs. **Table S3.** Gene Ontology enrichment among cis and distal eCpGs. **Table S4.** Gene Ontology enrichment among trans eCpGs. **Table S5.** Gene Ontology enrichment among trans eCpG associated transcripts. **Table S6.** Overlapping gene regulation. (DOCX 760 kb)

Additional file 6: Supplemental materials and methods. (DOCX 110 kb)
