# Supplementary material for: An integrated -omics analysis of the epigenetic landscape of gene expression in human blood cells
Source: BMC Genomics. 2018 Jun 19;19:476. doi: 10.1186/s12864-018-4842-3 (PMC6006777; doi:10.1186/s12864-018-4842-3)
Supplement: Supplementary file 5 — Figure S1. Distribution of methylation beta-value variances, plotted on a log scale. Figure S2. Distances between each CpG and the closest TSS for all CpG probes and expression probes included in GTP and MESA. Figure S3. Odds ratios for enrichment of chromatin features among CpGs. Figure S4. eCpG-transcript distance vs. HiC interaction frequency. Figure S5. Comparison of Genomic Inflation Factors obtained for GTP by the Houseman method and ICE. Table S1. Breakdown of eCpG-transcript status. Table S2. Gene Ontology enrichment among all eCpGs. Table S3. Gene Ontology enrichment among cis and distal eCpGs. Table S4. Gene Ontology enrichment among trans eCpGs. Table S5. Gene Ontology enrichment among trans eCpG associated transcripts. Table S6. Overlapping gene regulation. (DOCX 760 kb) [file 12864_2018_4842_MOESM5_ESM.docx]

**Supplemental Figures**


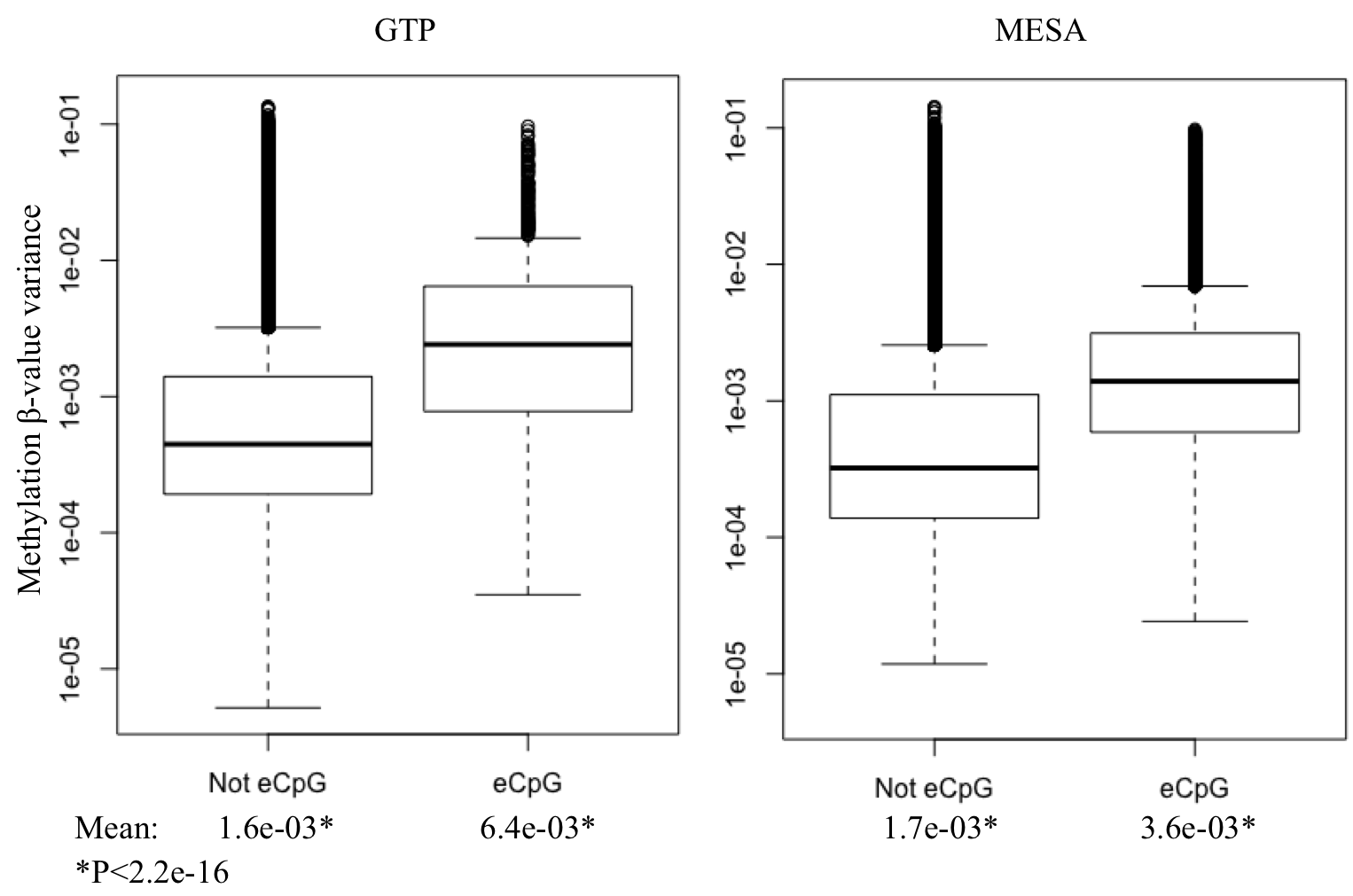


**Figure S1.** **Distribution of methylation beta-value variances, plotted on a log scale.** Variances for either expression-associated or not-expression-associated CpGs were calculated across samples for GTP and MESA. The results indicate that eCpGs have more variable beta-values across samples.


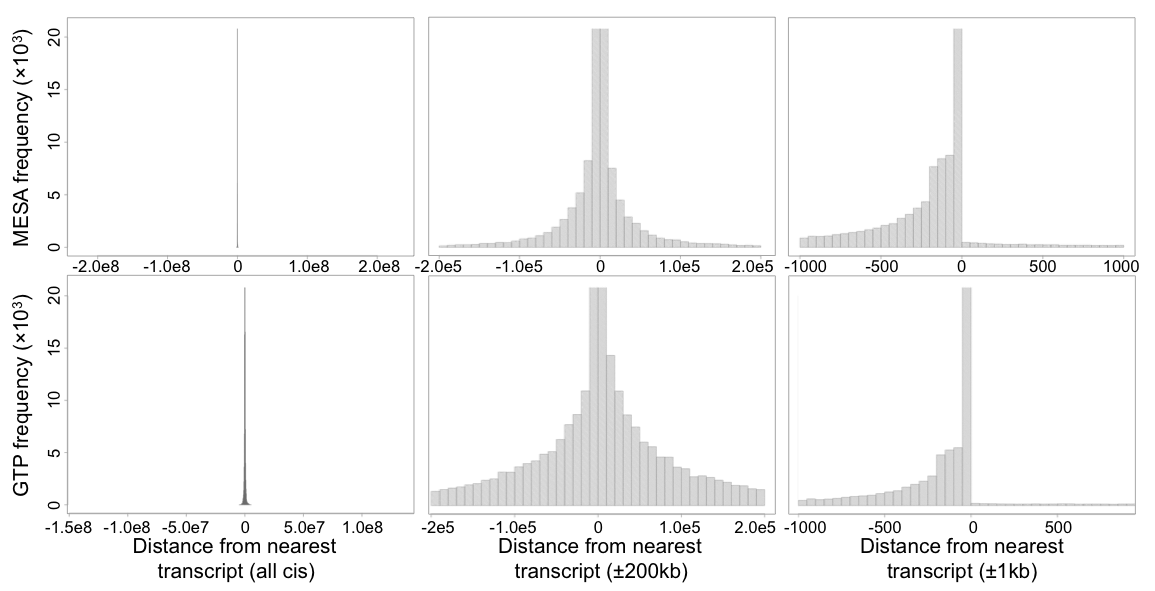


**Figure S2.** **Distances between each CpG and the closest TSS for all CpG probes and expression probes included in GTP and MESA.** Each panel contains the same data, focused on three different ranges of distances. The distribution of CpG-TSS distances for each array is similar to the distribution seen for eCpGs found in each study.


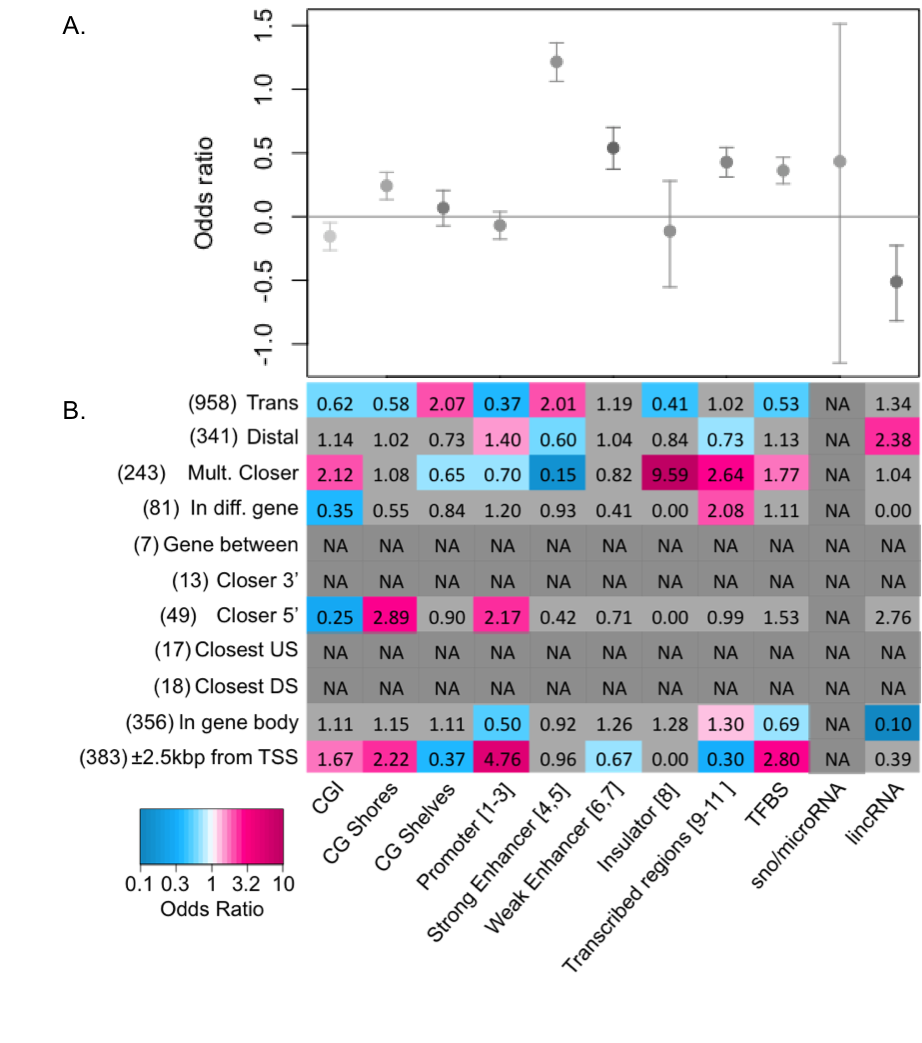


**Figure S3.** **Odds ratios for enrichment of chromatin features among CpGs. A)** Enrichment of eCpGs in the listed chromatin features, among all CpGs tested in GTP (N=472,199). **B)** Enrichment of eCpGs in the listed chromatin features, among genome-wide significant eCpGs in GTP (N=2,466). Blue indicates significant depletion and red, significant enrichment. Light gray cells were not significant and ORs in dark gray cells could not be estimated due to low counts. Bracketed numbers in the chromatin features indicate the ChromHMM state. Numbers in parentheses indicate the number of eCpGs in the category. **Definitions: Bottom.** “CGI” are CpG islands. “TFBS” is transcription factor binding site.


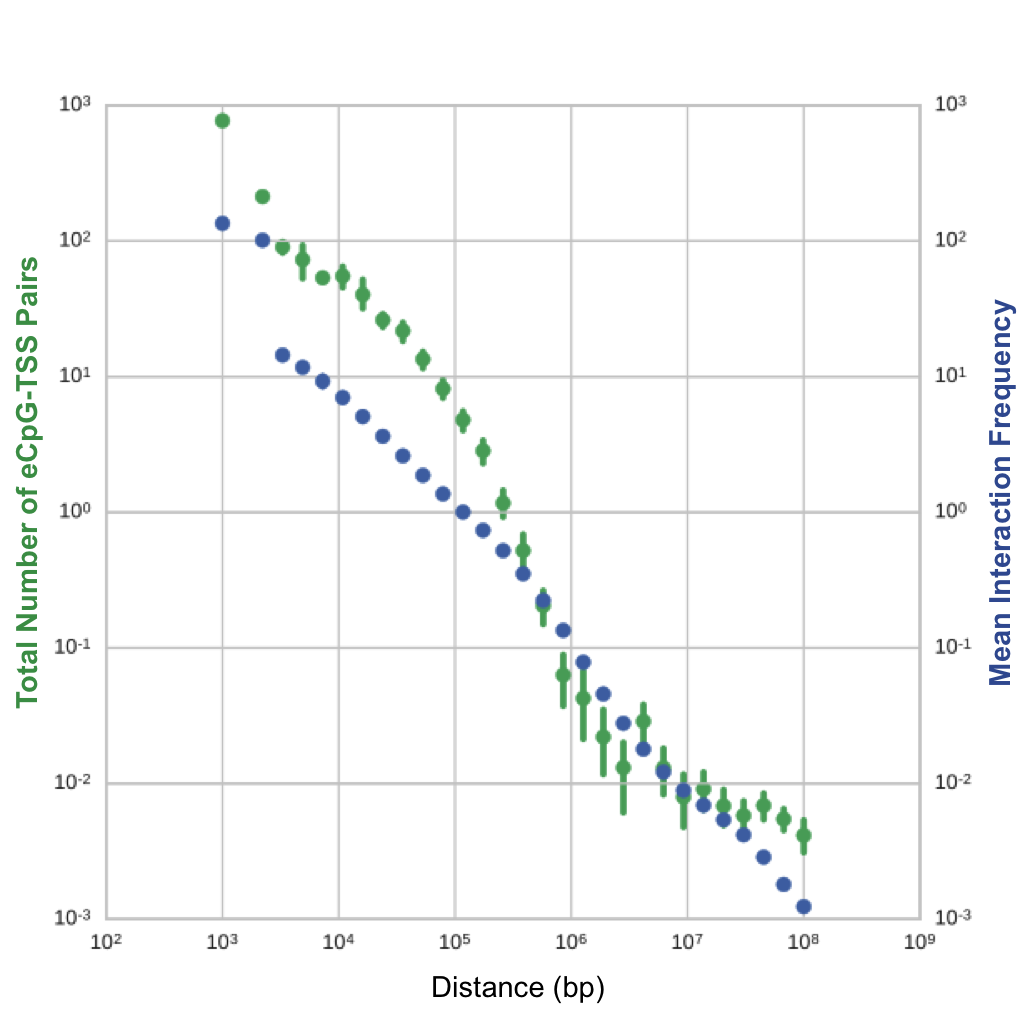


**Figure S4.** **eCpG-transcript distance vs. HiC interaction frequency.** Distribution of interactions over distance for MESA cis and distal eCpG-transcript pairs (green) and Hi-C interaction data from a lymphoblastoid cell line (GM12878; blue). Mean interaction frequency between each 1 kb bin (blue dots) were calculated for un-normalized Hi-C interaction counts on Chromosome 1. Number of eCpG-transcript pairs (green dots) per 1 kb were calculated by rounded distance between eCpG and TSS for cis and distal eCpG-transcript pairs. Bars represent 0.95 confidence intervals for each bin. The decay curve of eCpG-transcript pair distances within 10^6^ bp is consistent with the chromatin looping interaction curve seen in HiC.

**Figure S5. Comparison of Genomic Inflation Factors obtained for GTP by the Houseman method and ICE.** Genomic inflation factors (GIF) were calculated as median (T-statistic)^2^/0.4549 for each transcript. The Houseman method uses bioinformatically determined cell proportions as covariates in each regression to account for heterogeneity of cell types in epigenome wide association studies of methylation. The Intersample Correlation Emended method uses an intersample covariance matrix generated from transcript expression levels to account for unknown confounding.

Houseman method

Intersample

Correlation Emended


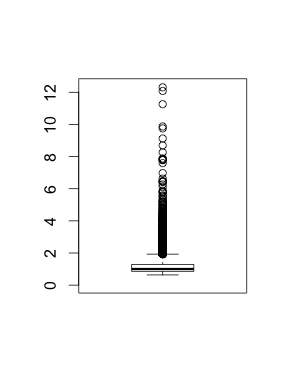

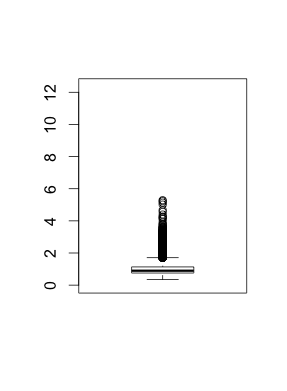


Genomic inflation factors

**Supplemental Tables**

| **Table S1. Breakdown of eCpG-transcript status** | | | | |
| --- | --- | --- | --- | --- |
| MESA | | | | |
| eCpG-transcript status | Counts | | Percent | |
| Cis | 7,246 | | 20.99 | |
| In gene body | 1,919 | | 26.48 |  |
| ± 2,500bp of TSS | 2,516 | | 34.72 |  |
| Closest upstream gene | 106 | | 1.46 |  |
| Closest downstream gene | 187 | | 2.58 |  |
| Multiple genes closer/between | 1,622 | | 22.38 |  |
| eCpG in different gene | 482 | | 6.65 |  |
| Gene between | 68 | | 0.94 |  |
| Closer 5’ | 301 | | 4.15 |  |
| Closer 3’ | 45 | | 0.62 |  |
| Distal | 3,460 | | 10.02 | |
| Trans | 23,812 | | 68.98 | |
| Total | 34,518 | |  | |
| GTP | | | | |
| eCpG-transcript status | Counts | Percent | | |
| Cis | 1,167 | 47.32 | | |
| In gene body | 356 | 30.51 | |  |
| ± 2,500bp of TSS | 383 | 32.82 | |  |
| Closest upstream gene | 17 | 1.46 | |  |
| Closest downstream gene | 18 | 1.54 | |  |
| Multiple genes closer/between | 243 | 20.82 | |  |
| eCpG in different gene | 81 | 6.94 | |  |
| Gene between | 7 | 0.60 | |  |
| Closer 5’ | 49 | 4.20 | |  |
| Closer 3’ | 13 | 1.11 | |  |
| Distal | 341 | 13.83 | | |
| Trans | 958 | 38.85 | | |
| Total | 2,466 |  | | |

| **Table S2. Gene Ontology enrichment among all eCpGs** | | | |
| --- | --- | --- | --- |
| Term (MF) | Odds Ratio | P-value | FDR |
| Sequence-specific DNA binding | 2.64 | 1.16E-04 | 3.23E-02 |
| Ribonucleoside binding | 1.45 | 1.17E-04 | 3.23E-02 |
| Purine nucleoside binding | 1.45 | 1.21E-04 | 3.23E-02 |
| Purine ribonucleotide binding | 1.43 | 1.72E-04 | 3.46E-02 |
| Binding | 1.51 | 1.94E-04 | 3.46E-02 |
| Transcription factor binding | 4.31 | 2.56E-04 | 3.92E-02 |

| **Table S3. Gene Ontology enrichment among cis and distal eCpGs** | | | |
| --- | --- | --- | --- |
| Term (MF) | Odds Ratio | P-value | FDR |
| Actin binding | 1.66 | 2.57E-05 | 1.94E-02 |
| Actin filament binding | 2.37 | 5.15E-05 | 1.94E-02 |
| Ankyrin binding | 10.35 | 5.26E-05 | 1.94E-02 |
| HMG box domain binding | 16.33 | 1.41E-04 | 3.91E-02 |
| Sequence-specific DNA binding | 1.48 | 1.80E-04 | 3.98E-02 |

| **Table S4. Gene Ontology enrichment among trans eCpGs** | | | |
| --- | --- | --- | --- |
| Term (MF) | Odds Ratio | P-value | FDR |
| Sequence-specific DNA binding | 2.99 | 4.56E-06 | 2.45E-03 |
| Ribonucleoside binding | 1.46 | 2.12E-05 | 5.75E-03 |
| Purine nucleoside binding | 1.46 | 2.19E-05 | 5.75E-03 |
| Purine ribonucleotide binding | 1.45 | 2.68E-05 | 5.75E-03 |
| Transcription factor binding | 4.22 | 6.37E-05 | 1.14E-02 |
| Ligase activity | 2.88 | 1.08E-04 | 1.66E-02 |
| Double-stranded DNA binding | Inf | 1.35E-04 | 1.71E-02 |
| Protein homodimerization activity | 1.79 | 1.43E-04 | 1.71E-02 |
| Adenyl nucleotide binding | 1.43 | 1.96E-04 | 2.11E-02 |
| Rab GTPase binding | Inf | 2.28E-04 | 2.22E-02 |
| Protein domain specific binding | 3.81 | 2.79E-04 | 2.32E-02 |
| ATP binding | 1.42 | 2.81E-04 | 2.32E-02 |
| Sequence-specific DNA binding transcription factor activity | 1.70 | 3.32E-04 | 2.55E-02 |
| Binding | 1.43 | 4.08E-04 | 2.92E-02 |
| Protein kinase binding | 2.23 | 5.58E-04 | 3.74E-02 |
| RNA polymerase II core promoter proximal region sequence-specific DNA binding | 3.58 | 5.99E-04 | 3.78E-02 |

| **Table S5. Gene Ontology enrichment among trans eCpG associated transcripts** | | | |
| --- | --- | --- | --- |
| Term (MF) | Odds Ratio | P-value | FDR |
| DNA binding | 1.39 | 1.08E-06 | 1.17E-04 |
| Single-stranded DNA binding | 3.33 | 3.02E-06 | 2.94E-04 |
| Hydrolase activity, acting on acid anhydrides | 1.50 | 4.22E-06 | 3.74E-04 |
| Structural constituent of ribosome | 2.52 | 6.54E-06 | 5.32E-04 |
| Ubiquitin-protein transferase activity | 1.82 | 1.34E-05 | 9.50E-04 |
| Chromatin binding | 1.71 | 1.36E-05 | 9.50E-04 |
| Ligase activity | 1.73 | 6.21E-05 | 4.03E-03 |
| Ubiquitin binding | 3.73 | 1.43E-04 | 8.70E-03 |
| ATP-dependent helicase activity | 3.86 | 1.60E-04 | 9.16E-03 |
| Helicase activity | 2.47 | 2.31E-04 | 1.25E-02 |
| Histone acetyltransferase activity | 3.78 | 3.92E-04 | 2.01E-02 |
| RNA binding | 1.68 | 5.66E-04 | 2.66E-02 |
| Nucleosomal DNA binding | 4.32 | 5.72E-04 | 2.66E-02 |
| Protein transporter activity | 2.48 | 6.15E-04 | 2.66E-02 |
| Histone deacetylase binding | 2.39 | 6.27E-04 | 2.66E-02 |
| Protein C-terminus binding | 1.74 | 1.07E-03 | 4.35E-02 |
| Ligand-dependent nuclear receptor transcription coactivator activity | 2.85 | 1.17E-03 | 4.57E-02 |

| **Table S6. Overlapping gene regulation** | | | | | | | |
| --- | --- | --- | --- | --- | --- | --- | --- |
| First gene | eCpG location | Strand | Corr | Second gene | eCpG location | Strand | Corr |
| TYMP | Gene body | - | + | SCO2 | Promoter | - | - |
| TMIGD3 | Gene body | - | + | ADORA3 | Promoter | - | - |
| HLA-DPA1 | Gene body | - | + | HLA-DPB1 | Promoter | + | - |
| KRT10 | Gene body | + | - | TMEM99 | Promoter | + | - |
| GFM1 | Gene body | + | - | LXN | Promoter | - | - |
