# Supplementary material for: An integrated -omics analysis of the epigenetic landscape of gene expression in human blood cells
Source: BMC Genomics. 2018 Jun 19;19:476. doi: 10.1186/s12864-018-4842-3 (PMC6006777; doi:10.1186/s12864-018-4842-3)
Supplement: Supplementary file 6 — Supplemental materials and methods. (DOCX 110 kb) [file 12864_2018_4842_MOESM6_ESM.docx]

**Supplemental Methods**

**Study Populations**

*The Grady Trauma Project (GTP)*

GTP is a prospective study of stress-related outcomes in which participants were recruited from the waiting rooms of Grady Memorial Hospital’s General Practice or Obstetrics and Gynecology departments. Participants are from an inner city population with higher than average rates of trauma exposure and post-traumatic stress disorder, but are representative of this population in that they are not specifically ascertained for presence of disease or trauma. Exclusion criteria included mental retardation, active psychosis, or the inability to give written and verbal informed consent. The Institutional Review Boards of Emory University School of Medicine and Grady Memorial Hospital approved all procedures in this study. Genome-wide DNA methylation and gene expression measurements were generated for 333 human blood samples collected in Atlanta, Georgia as part of the Grady Trauma Project [1]. GTP participants included in this study range between 18 and 78 years old. 76% are female and all are African-American, based on self report and confirmation via principal component analysis of genotype data [2].

For gene expression analysis, whole blood was collected in Tempus RNA tubes. All whole genome expression profiles were generated at the Max-Planck Institute. RNA was isolated using the Versagene kit (Gentra Systems, Minneapolis, U.S.A.) and quantified using the Nanophotometer (Implen, München, Germany). Quality checks were performed on the Agilent Bioanalyzer. 250 nanograms of RNA were reverse transcribed to cDNA, converted to cRNA and biotin-labeled using the Ambion kit (AMIL1791, Applied Biosystems). 750 nanograms of cRNA were hybridized to Illumina HT-12 v3.0 or v4.0 arrays (Illumina, San Diego, California, U.S.A) and incubated for 16 hours at 55ºC. Arrays were then washed, stained with Cy3 labeled streptavidin, dried and scanned on the Illumina BeadScan confocal laser scanner. Expression values were normalized using the variance stabilizing transformation. 13,933 transcripts from the v3.0 and v4.0 arrays and were significantly expressed above background levels (detection P<0.01) in at least 5% of subjects, and were used in further analysis.

We compared the average log expression for probes common to both array versions between the samples run on the v3 and v4 arrays. We found that the average log expression was highly (0.9999) correlated between the two array versions. In the figure below, the axes are the average log expression at one transcript across samples processed on either the v3 (x) or v4 (y) array. Each circle is one transcript that is common to both arrays.


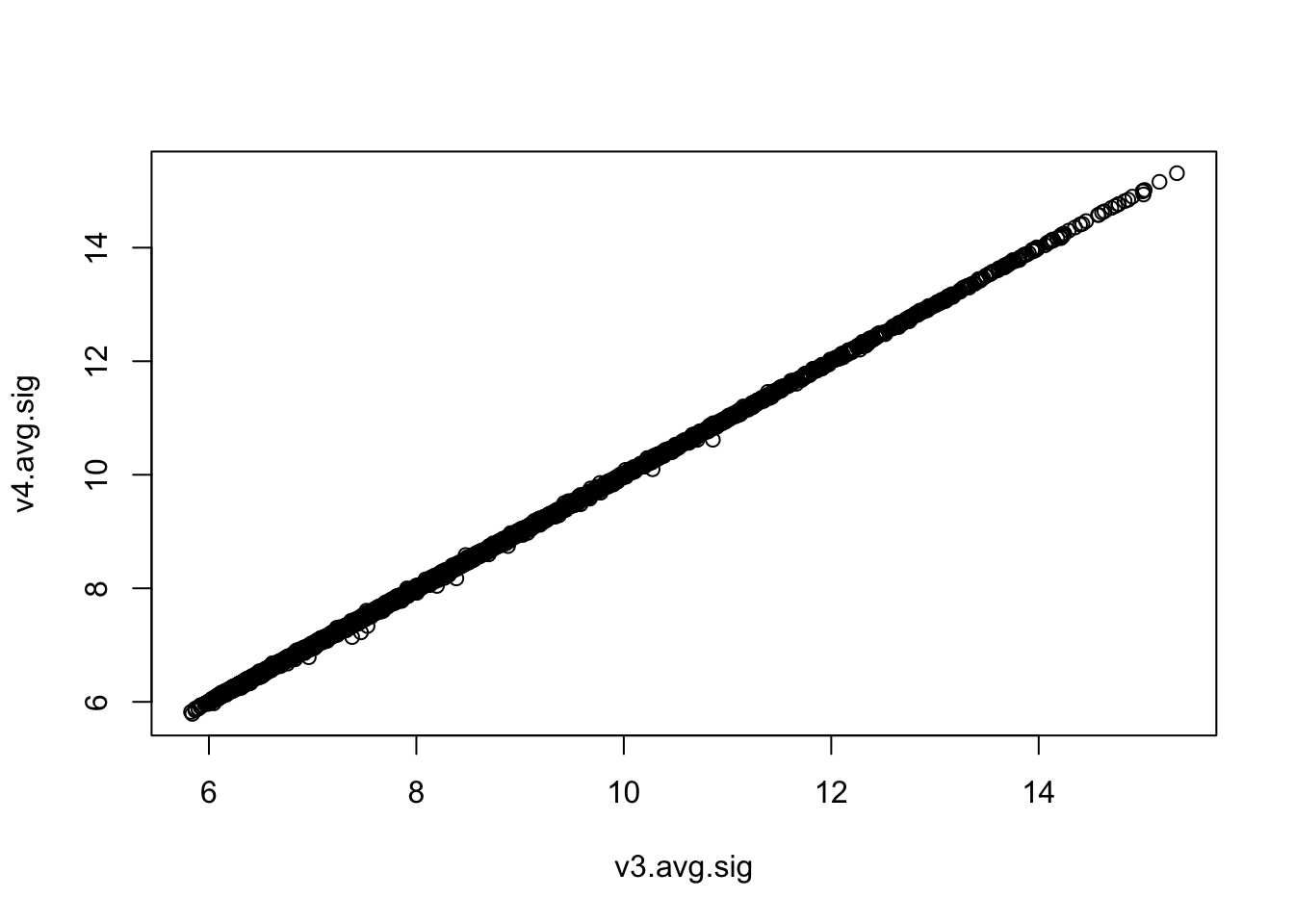


DNA was extracted from whole blood at the Max-Planck Institute in Munich using the Gentra Puregene Kit (Qiagen), to assay CpG methylation. Genomic DNA was bisulfite converted using the Zymo EZ-96 DNA Methylation Kit (Zymo Research) and applied to the Illumina HumanMethylation450 BeadChip, with hybridization and processing performed according to the instructions of the manufacturer. Methylated (M) and unmethylated (U) signals were collected for all CpG sites on the array and quantile normalized across all samples. β-values for each individual at each CpG site were calculated as the total methylated signal divided by the total signal (M/M+U). Data points with 1) a detection p-value greater than 0.001 or 2) a combined signal less than 25% of the total median signal and less than both the median unmethylated and median methylated signal were set to missing. CpG sites with a missingness rate above 10% were removed from analysis. Individual samples were removed from analysis if they were outliers in a hierarchical clustering analysis or had 1) a mean total signal less than half of the median overall mean signal or 2000 arbitrary units, or 2) a missingness rate above 5%.

*Multi-ethnic Study of Atherosclerosis (MESA)*

MESA DNA methylation and gene expression data were collected from the Gene Expression Omnibus (GSE56047). The following summarizes the methods detailed in Liu et al. (2013).

MESA is a study designed to examine cardiovascular disease. The MESA Epigenomics and Transcriptomics Study investigates gene expression regulatory methylation sites in humans by examining the association between CpG methylation and gene expression in purified human monocytes from a large study population. Genome-wide DNA methylation and gene expression measurements were generated for purified monocytes from 1,202 MESA participants. These MESA participants were chosen randomly from samples collected between April 2010 and February 2012 from MESA field centers in Baltimore, MD; Forsyth County, NC; New York, NY; and St Paul, MN. Participants range in age from 55 – 94 years old, are 51% female, and self identified as Caucasian (47%), African American (21%), or Hispanic (32%).

Monocytes were isolated from PBMCs with anti-CD14 coated magnetic beads. DNA and RNA were isolated from monocyte samples simultaneously. DNA and RNA purity were assessed spectrophotometrically and RNA QC testing was performed using the Agilent 2100 Bioanalyzer with RNA 6000 Nano chips (Agilent Techonology, Inc., Santa Clara, CA) according to the manufacturer’s instructions. Samples with RIN (RNA Integrity) scores > 9.0 were applied to global gene expression microarrays.

Genome-wide expression analysis was performed via the Illumina HumanHT-12 v4 Expression BeadChip and the Illumina Bead Array Reader, following the Illumina expression protocol. RNA was reverse transcribed and amplified with the Illumina TotalPrep-96 RNA Amplification Kit (Ambion/Applied Biosystems, Darmstadt, Germany). The resulting cRNA was hybridized to a BeadChip (CITATION).

Expression data were corrected for local background in Illumina’s proprietary software GenomeStudio and negative controls were used to compute detection P-values. Normal-exponential convolution model analysis was used to estimate non-negative signal. All probes and samples were quantile normalized, offset and log_2_ transformed [3]. Expression probes with detection p-values >0.01 in at least 5% of samples were removed.

Bead-level methylation data were summarized in and collected from GenomeStudio. Methylation data were smooth quantile normalization (to adjust for color bias) normalized, background corrected (by subtracting the median intensity of the negative control probes), and quantile normalized across all samples with the R package, lumi. [3]. The final methylation value for each methylation probe was computed as the M-value [M is logit(beta-value)]. We collected these values from GEO, transformed the *M*-values to β-values and performed additional QC (concerning missingness and low signal intensity; as in the GTP methylation data).

**Transcript Annotation.** The Illumina HT12 probe locations were provided a re-annotation of the array (Bioconductor packages illuminaHumanv3.db and illuminaHumanv4.db; Barbosa-Morais et al., 2010). Probes annotated as “bad” or “’no match” were removed from analysis. Each probe was assigned multiple genomic locations, based on the “GenomicLocation”, “SecondMatches”, and “OtherGenomicMatches” listed in the re-annotation. Refseq and Ensembl transcript and exon intervals for the HG19 build were collected from the UCSC table browser. When exons for the same gene overlapped, the start and end points yielding the largest interval were taken to make a representative or average exon. Refseq and Ensembl gene information was annotated to each expression probe ID (using R Bioconductor package GenomicRanges; [5] if the probe interval overlapped the exon interval by more than 25 bp. Where more than one gene exon overlapped the expression probe, poorly characterized genes (gene names beginning with KIAA, FLJ or LOC and those containing “orf”) and read-through transcripts were removed from the duplicates. The remaining duplicate gene names were compiled for that probe ID and the transcript area taken as the largest interval formed by the overlapping transcripts. Similarly, if overlapping transcript areas (transcription start site to transcription end site, including introns) existed for the same gene name in the Refseq or Ensembl tables, the minimum TSS and maximum TES were taken for the gene entry. Refseq or Ensembl tables with representative transcript intervals are referred to as average Refseq and average Ensembl gene locations in the Materials and Methods section.

**Assignment of Probe location.** For each eCpG-transcript pair, all probe locations (for one Probe ID) were compared to the eCpG location. 3,943 (29.2%) of the expression probes passing QC in GTP and 6,335 (32.6%) of the expression probes passing QC in MESA had multiple possible genomic locations. Probe locations were prioritized in the following order:

1. The eCpG location fell within the gene annotated to one of the probe locations or up to 2,500 bp upstream of that gene’s TSS.
2. The eCpG was within 1 megabase of the TSS of the gene annotated to one the probe locations.
3. The eCpG was on the same chromosome as one of the probe locations.

If more than one probe location fell into the highest priority group, they were further filtered by the following criteria until one probe location was chosen: locations annotated to a gene are preferred, locations marked as GenomicLocation > SecondMatches > OtherGenomicMatches, locations in which the eCpG was closest to the TSS of the annotated gene were preferred. If all probe locations were on different chromosomes than the eCpG, probe locations were chosen as follows: locations annotated to a gene are preferred, locations marked as GenomicLocation > SecondMatches > OtherGenomicMatches. The effects of these decisions are outlined in the following table, which compares the proportions of cis, distal and trans eCpGs in MESA and GTP both before and after the implementation of the above probe assignment rules.

| **Comparison of status proportions before and after implementation of flexible expression probe assignment** | | |
| --- | --- | --- |
| **MESA** | | |
| **eCpG-transcript status** | **Before (%)** | **After (%)** |
| **Cis** | **19.9** | **21.0** |
| **Distal** | **9.4** | **10.0** |
| **Trans** | **70.7** | **69.0** |
| **GTP** | | |
| **eCpG-transcript status** | **Before** | **After** |
| **Cis** | **39.4** | **47.3** |
| **Distal** | **11.1** | **13.8** |
| **Trans** | **49.4** | **38.9** |

**Genomic Interaction Distance Decay**

In this analysis we used the GM12878 Hi-C dataset of Rao et. al 2014 accessed from GSE63525 [28]. The average number of interactions between each 1 kb bin was taken from the expected values for unnormalized interaction counts on Chromosome 1 at 1 kb. We next calculated the distance (rounded to the nearest 1 kb) between eCpGs and the transcription start sites (TSS) annotated to their associated expression probes for each of the 4,799 significant cis and distal eCpG-transcript pairs. To plot the distance decay of both datasets, the logarithmic space between 10^3^ and 10^8^ was divided into 30 equally spaced bins. Means and 0.95 confidence intervals were computed for each bin using the .regplot function in the python package, Seaborn (DOI 10.5281/zenodo.592845).

**Further Gene Body eCpG Analysis**

To test the hypothesis that positively correlated gene body eCpGs reside in the promoters of overlapping genes, we first identified eCpGs within our results that were associated with expression transcripts that annotated to overlapping genes. Read-through genes and low-confidence (i.e., LOC, orf, KIAA, FLJ) annotations were excluded. We compared the refseq and ensembl annotations separately. From these eCpGs we were able to compare the numbers of negative and positive eCpG-gene body correlations.

1. Gillespie CF, Bradley B, Mercer K, Smith AK, Conneely K, Gapen M, et al. Trauma Exposure and Stress-Related Disorders in Inner City Primary Care Patients. Gen Hosp Psychiatry. 2009;31:505–14.

2. Kilaru V, Iyer SV, Almli LM, Stevens JS, Lori A, Jovanovic T, et al. Genome-wide gene-based analysis suggests an association between Neuroligin 1 (NLGN1) and post-traumatic stress disorder. Transl Psychiatry. 2016;6:e820.

3. Liu Y, Ding J, Reynolds LM, Lohman K, Register TC, Fuente ADL, et al. Methylomics of gene expression in human monocytes. Hum Mol Genet. 2013;:ddt356.

4. Barbosa-Morais NL, Dunning MJ, Samarajiwa SA, Darot JFJ, Ritchie ME, Lynch AG, et al. A re-annotation pipeline for Illumina BeadArrays: improving the interpretation of gene expression data. Nucleic Acids Res. 2010;38:e17–e17.

5. Lawrence M, Huber W, Pagès H, Aboyoun P, Carlson M, Gentleman R, et al. Software for Computing and Annotating Genomic Ranges. PLoS Comput Biol. 2013;9:e1003118.
